# Health behaviors among college students: the influence of future time perspective and basic psychological need satisfaction

**DOI:** 10.1080/21642850.2013.872992

**Published:** 2014-01-21

**Authors:** Preston L. Visser, Jameson K. Hirsch

**Affiliations:** ^a^ Lawndale Christian Health Center, East Tennessee State University, Johnson City, TN37614, USA; ^b^ Department of Psychology, East Tennessee State University, Johnson City, TN37614, USA

**Keywords:** self-determination theory, basic psychological needs, future time perspective, health behaviors

## Abstract

Health behavior change may prevent many fatal diseases, and may be influenced by social and motivational constructs. We assessed the interaction effect of future time perspective and basic psychological need fulfillment on positive and negative health behaviors. Future time perspective was associated with more positive, and less negative, health behaviors. Need fulfillment was associated with only positive health behaviors. In moderation analyses, individuals reporting both high need fulfillment and future perspective reported greater positive health behaviors, and were especially unlikely to smoke. Enhancing future-mindedness and supporting need satisfaction in interventions targeting modifiable health behaviors is encouraged.

## 1. Introduction

Modifiable negative health behaviors, including smoking, poor diet, and physical inactivity, are leading causes of death for people in the USA (Mokdad, Marks, Stroup, & Gerberding, 2004). In general, negative health behaviors lead to immediate or long-term deleterious health consequences, whereas positive health behaviors serve to preserve or improve health status (Henson, Carey, Carey, & Maisto, 2006). Of importance, the majority of individuals in college maintain an unhealthy diet (Racette, Deusinger, Strube, Highstein, & Deusinger, 2005) and the 12-month prevalence of tobacco use among college students is nearly 50%, with almost a third reporting current use (Rigotti, Lee, & Wechsler, 2000). Young adults are also at an increasing risk for medical problems such as type 2 diabetes and coronary artery disease due to declines in health behaviors, such as diet and exercise (Desai, Miller, Staples, & Bravender, 2008), and health behavior patterns established at a younger age are likely to persist throughout adulthood (DiLorenzo, Stucky-Ropp, Vander Wal, & Gotham, 1998).

As such, focused public health interventions targeting changes in health behaviors are needed, before health complications arise (US Department of Health and Human Services, 2000). To be effective, however, health behavior interventions must target modifiable, motivational characteristics associated with the engagement in positive and avoidance of negative health behaviors (Henson et al., 2006; Yarcheski, Mahon, Yarcheski, & Cannella, 2004). Two such constructs are time perspective and self-determination. Time perspective refers to temporal orientation and is thought to guide general, as well as health-related, decision-making and behavior (Henson et al., 2006; Mahon, Yarcheski, & Yarcheski, 1997; Zimbardo & Boyd, 1999). Self-determination is a motivational process involving a striving to satisfy basic psychological needs (BPNs) and a tendency toward pursuit of behaviors that lead to healthy and adaptive functioning (Ryan & Deci, 2000; Ryan, Williams, Patrick, & Deci, 2009). The potential importance of these constructs for health behaviors is well-established, yet they have rarely been examined simultaneously (Miller & Brickman, 2004), as we do in the current study.

When thinking about the future, humans are capable of engaging in abstract cognitive processes in which they draw upon past experience and weigh competing options in the present to construct possible futures (Zimbardo & Boyd, 1999). Individuals adhering to future time perspective tend to persistently engage in future thinking and planning, with the intent of ensuring that their present behaviors align with future aspirations. Future time perspective has been positively associated with health-related protective behaviors such as exercise and the use of condoms and seat belts, and with fewer health-related risk behaviors such as tobacco and drug use (Hall & Fong, 2003; Henson et al., 2006; Mahon et al., 1997). Younger adults often have more difficulty envisioning the future and, consequently, enacting health behaviors to achieve lifespan health goals (Hoppmann & Blanchard-Fields, 2010); however, adopting a future time perspective early in life may be especially important in successfully establishing healthy behavioral patterns that persist throughout adulthood and into older age (Rakowski, 1986).

With regard to BPNs, the self-determination theory (SDT), a macro-theory of human motivation, emphasizes that people have an innate tendency toward constructive growth and integration of new experiences into a coherent sense of self (Ryan & Deci, 2000). Social factors can inhibit or support natural tendencies toward psychological growth and active engagement, particularly as they impact what SDT labels the three universal BPNs: autonomy, or a sense of choicefulness and ownership in one's behavior; competence, or the sense that one can successfully act upon and affect the environment in a desired manner; and relatedness, or feelings of belongingness and interpersonal connection (Deci & Ryan, 2008; Ryan & Brown, 2003). Autonomy is supported when people are not coerced but, rather, are able to choose their behavior freely; competence is supported when individuals work toward difficult yet achievable goals and receive relevant positive feedback; and relatedness is supported by the receipt of perceived acceptance and caring from others (Ryan & Brown, 2003). The SDT posits that intrinsic motivation stems from individual fulfillment of BPNs. Therefore, individuals whose BPNs are generally fulfilled are more likely to act in self-determined and beneficial ways, whereas those whose basic needs are generally thwarted may seek to compensate for their lack of fulfillment by engaging in externally motivated and detrimental behavior (Ryan & Deci, 2000).

Prior research has related basic psychological need fulfillment to self-regulation toward beneficial behaviors like exercise and healthy dieting (Edmunds, Ntoumanis, & Duda, 2006; Fortier, Sweet, O'Sullivan, & Williams, 2007; Ng et al., 2012; Vlachopolous & Michailidou, 2006). Autonomy and competence appear to play central roles in physical activities such as exercise, whereas relatedness contributes to motivation for overall health and well-being across the lifespan (Ryan & Deci, 2000; Wilson & Todd Rogers, 2008).

The functioning of personality is complex and, as such, characteristics such as future time perspective and PBNs may combine, perhaps synergistically, to predict health behaviors. Individuals with high basic need satisfaction may have stronger motivation to engage in healthy behaviors, but may fail to act accordingly for a variety of reasons, such as distraction and neglecting to plan for obstacles (Brown, 2003; Wilson & Todd Rogers, 2008). Future orientation, in addition to motivation, may help one circumvent obstacles to healthy behavior changes (Adams & White, 2009; Chang et al., 2013). Conversely, one might possess strong future orientation yet lack persistent motivation. Ryan and Deci (2000) argue that *future* goals satisfying BPNs stem from “authentic” or “self-determined” motivation, whereas goals satisfying external pressures stem from “controlled” motivation. The former scenario involves more interest, confidence, and persistence in goal pursuit than the latter. Therefore, we expect that participants reporting both higher levels of basic psychological need fulfillment and greater future time perspective will also report more positive and less negative health behaviors.

Other researchers have examined the constructs of self-determination and future orientation simultaneously in the context of education and exercise (Miller & Brickman, 2004; Tabachnick, Miller, & Relyea, 2008; Vansteenkiste, Simons, Lens, Sheldon, & Deci, 2004). Although they highlight the importance of future orientation and basic psychological need fulfillment for adaptive behavior, these previous studies primarily investigate the social contextual factors that influence the motivation and behavior (e.g. framing effects). No known studies have examined the possible interactions between the individual difference factors of general basic psychological need fulfillment and future time orientation, as we do in the current study.

We hypothesized that future time perspective and fulfillment of BPNs, including overall and specific needs, would be positively associated with positive health behaviors and negatively associated with negative health behaviors. We also hypothesized that future time perspective would moderate the association between BPNs and health behaviors, such that individuals reporting greater future time perspective would endorse higher levels of positive, and less negative, health behaviors associated with Basic Psychological Needs Scale (BPNS) total and subscale scores, whereas those with a weaker future time perspective would report less positive and more negative health behaviors. Because BPNs are non-independent, meaning they co-occur as motivators, we examined our hypotheses in both independent and a combined model with all basic psychological need subscales and interactions.

## 2. Methods

### 2.1. Participants

Participants were 439 students enrolled in an undergraduate psychology course at a regional university in the southeastern USA. In our Institutional Review Board-approved study, participants completed an informed consent procedure, then completed an online survey, and were awarded research credit for their participation. Females comprised 70.82% (*n* = 311) of the sample and the majority (91.34%; *n* = 401) of students reported that they consider themselves to be White. The mean age of the sample was 21.02 years (SD = 6.10; median = 19) and most students were either first- (45.79%; *n* = 201) or second-year students (20.96%, *n* = 92).

### 2.2. Measures

We assessed *future time perspective* using the Zimbardo Time Perspective Inventory (ZTPI), which is a 56-item instrument with 5 factors reflecting how individuals perceive time (Zimbardo & Boyd, 1999). The ZTPI subscale of future time perspective (FTP; 13 items), the only dimension used in the current analyses, assesses orientation to future occurrences. An example of FTP item is “When I want to achieve something, I set goals and consider specific means for achieving those goals.” In past research with college students, the ZTPI FTP demonstrated excellent internal consistency and test–retest reliability and validity was supported with expected associations with health behaviors such as smoking and exercise (Adams & Nettle, 2009; Hamilton, Kives, Micevski, & Grace, 2003; MacKillop, Anderson, Castelda, Mattson, & Donovick, 2006; Zimbardo & Boyd, 1999). In the current study, Cronbach's *α* = .83.

*BPNs* were assessed using the BPNS, a 21-item instrument assessing the extent of perceived fulfillment of autonomy, competence, and relatedness (Deci & Ryan, 2000). The BPNS yields a total score, as well as subscale scores for each need. Example items include, “I generally feel free to express my ideas and opinions” (autonomy), “Most days I feel a sense of accomplishment from what I do” (competence), and “People in my life care about me” (relatedness). In university samples, internal consistency was excellent for the total BPNS and acceptable for the subscales (Faye & Sharpe, 2008; Gagne, 2003). In the current sample, *α* was .71 for autonomy, .71 for competence, .83 for relatedness, and .89 for the total BPNS score.

*Positive and negative health behaviors* were assessed using the Multidimensional Health Profile–Health Functioning scale (MHP-H), a 69-item assessment of health behaviors, attitudes, and beliefs (Karoly, Ruehlman, & Lanyon, 2005). Examples of positive health behaviors include walking and tracking caloric intake and examples of negative health behaviors include eating sweets and smoking tobacco. In a national sample of adults, test–retest reliability of the health behaviors scale was good and validity was supported with expected associations with other health measures (Karoly et al., 2005). In the current study, internal consistency estimates for the two subscales were adequate: *α* = .69 for positive health behaviors and .65 for negative health behaviors; however, it is not expected that such checklist accounts of potentially exclusive behaviors would exhibit high levels of internal consistency.

### 2.3. Procedures and statistical analyses

In this Institutional Review Board-approved study, we used a secure online survey program to collect data across three semesters from students who participated for extra course credit. Pearson's product moment correlation coefficients were used to examine associations among study variables and hierarchical, multivariable linear regressions were used to assess moderation. Analytic models were constructed to assess the overall effect of BPNs (total score) and the independent effects of each BPNS subscale (autonomy, competence, and relatedness), in separate analyses. BPNS subscales were also examined in a combined model, simultaneously; covariates included age, gender, and ethnicity. Predictors were centered prior to creating interaction terms and, along with covariates, were entered on the first step of regression models and interaction terms were entered on the second step. Data were analyzed using IBM SPSS v20.

## 3. Results

As expected, higher FTP was associated with more positive health behaviors and fewer negative health behaviors. Additionally, higher scores on the total BPNS and each subscale were significantly associated with more positive health behaviors, but none of the BPNS was associated with negative health behaviors (Table 1).

**Table 1.** Means, standard deviations, and intercorrelations for study variables.

Variable	Mean (SD) *N* (%)	Sex	Age	FTP	Autonomy	Competence	Relatedness	BPNS total	Positive health behaviors	Negative health behaviors
Sex (female)	311 (71%)	–	–	–	–	–	–	–	–	–
Age	21.02 (6.10)	.01	–	–	–	–	–		–	–
FTP	3.59 (.61)	.21***	.12*	–	–	–	–	–	–	–
Autonomy	4.93 (.89)	.10*	.03	.15**	–	–	–	–	–	–
Competence	5.08 (.99)	.04	.05	.28***	.66***	–	–	–	–	–
Relatedness	5.50 (.99)	.07	−.03	.18***	.60***	.64***	–	–	–	–
BPNs total	15.51 (2.50)	.08	.02	.23***	.86***	.89***	.86***	–	–	–
Positive health behaviors	43.43 (6.74)	.14**	.08	.24***	.14**	.15**	.12*	.16**	–	–
Negative health behaviors	27.25 (5.20)	−.05	−.02	−.26***	.04	.03	.01	.03	.05	–
Smoking	2.13 (1.12)	−.08	.05	−.22**	−.05	−.04	−.08	−.07	−.13**	.56***

Notes: *N* = 439. FTP = ZTPI, Future subscale; Autonomy, Competence, Relatedness, and BPNs Total = BPNS; and positive health behaviors, negative health behaviors, and Smoking = MHP–Health Behaviors subscales.

*p ≤ .05.

**p ≤ .01.

***p ≤ .001.

To assess the FTP as a moderator of total BPNS and health behaviors, we conducted a series of hierarchical multivariable regressions (Table 2). The total BPNS score was significantly associated with positive health behaviors (*t* = 2.53, *p* < .05; Un *β* = .33, Standard Error (SE) = .19) and the interaction term between FTP and BPNS total score was significant (*t* = 2.34, *p* < .05; Un *β* = .44, SE = .19) such that individuals reporting both greater FTP and BPNS were more likely to endorse engagement in positive health behaviors.

**Table 2.** Moderation analyses: FTP × BPNs total score predicting health behaviors.

	Positive health behaviors		Negative health behaviors		Smoking	
	*t*-Value	*Β*	*t*-Value	*β*	*t*-Value	*β*
Step one						
Constant	33.46***		28.46***		6.51***	
Gender	2.03*	.10	0.07	.00	−0.62	−.03
Age	0.89	.04	0.80	.04	2.09*	.10
Ethnicity	1.40	.07	−3.43**	−.16	−2.26*	−.11
FTP	3.81***	.19	−5.98***	−.29	−4.45***	−.22
BPN	2.23*	.11	1.98*	.09	−0.38	−.02
Step two						
Constant	33.63***		28.46***		6.53	
Gender	2.03*	.10	0.08	.00	−0.61	−.03
Age	0.78	.04	0.86	.04	2.22*	.11
Ethnicity	1.32	.06	−3.39**	−.16	−2.19*	−.10
FTP	3.98***	.19	−6.04***	−.29	−4.61***	−.23
BPN	2.53*	.12	1.83	.09	−0.70	−.03
FTP × BPN	2.34*	.11	−1.03	−.05	−2.23*	−.11

Notes: *N* = 439. Positive and Negative health behaviors and Smoking = MHP; FTP = ZTPI – Future Time subscale; and BPNS = BPNS total score. Positive health behaviors *R* ^2^ = .08, Δ*R* ^2^ = .01; Negative health behaviors *R* ^2^ = .10, Δ*R* ^2^ = .01; and Smoking *R* ^2^ = .06, Δ*R* ^2^ = .01.

*p ≤ .05.

**p ≤ .01.

***p ≤ .001.

We also conducted independent regressions utilizing each of the three BPNs. Consistent with expectations, autonomy predicted higher positive health behaviors (*t* = 2.16, *p* < .05; Un *β* = .76, SE = .35) and the interaction of autonomy and FTP was significant (*t* = 2.01, *p* < .05; Un *β* = 1.02, SE = .51). Individuals with greater autonomy, as well as greater FTP, endorsed more positive health behaviors. Similarly, relatedness was associated with greater positive health behaviors (*t* = 2.43, *p* < .05; Un *β* = .78, SE = .32) and FTP moderated this association (*t* = 3.14, *p* < .01; Un *β* = 1.49, SE = .47) such that individuals with greater FTP reported more positive health behaviors associated with relatedness.

To examine their relative importance, we examined the combined contributions of all BPNS subscales in a single analytic model. Only the interaction between relatedness and FTP was significant (*t* = 2.85, *p* < .01; Un *β* = 1.80, SE = .63); at higher levels of future orientation, relatedness satisfaction and positive health behaviors were more strongly associated.

In analyses examining the association of BPNs with negative health behaviors, no interaction between BPNS total or subscale score and FTP was significant, including when BPNS subscales were examined independently or together in the same analytic model. In exploratory post-hoc analyses, we conducted a series of moderation tests to examine each of the negative health behaviors individually. We found significant interactions when smoking “cigarettes, cigars, or a pipe” was included as the dependent variable. FTP was a significant moderator of the associations between total BPNs score and smoking (*t* = −2.23, *p* < .05; Un *β* = −.08, SE = .04), autonomy and smoking (*t* = −2.00, *p* < .05; Un *β* = −.19, SE = .10), and relatedness and smoking (*t* = −2.93, *p* < .01; Un *β* = −.26, SE = .09). In each case, those individuals reporting higher levels of basic psychological need satisfaction and FTP were especially unlikely to report smoking. Main effects also existed in each model for FTP, which was negatively associated with smoking (*p* < .001).

**Figure 1.**
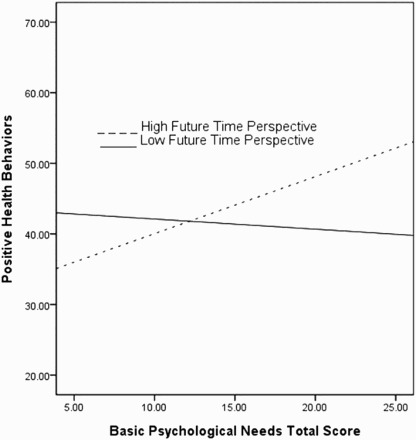
Interaction between time perspective, basic psychological needs and health behaviors.Notes: Positive health behaviors = MHP subscale score; FTP = ZTPI subscale (±1 SD); and BPNs total score = BPNS.

**Figure 2.**
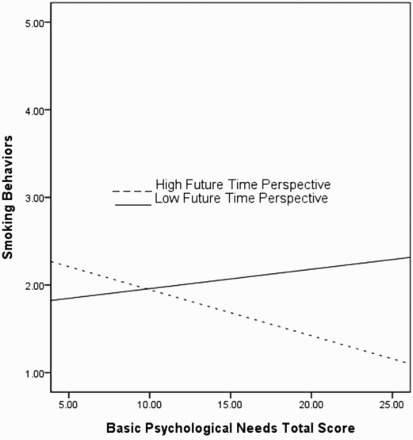
Interaction between time perspective, basic psychological needs and smoking behaviors.Notes: Smoking behaviors = MHP subscale item score; FTP = ZTPI subscale (±1 SD); and BPNs total score = BPNS.

Finally, we examined the relative importance of each BPNS subscale and its interaction with FTP for smoking behaviors. When entered into a single analytic model, only relatedness interacted with FTP to predict reduced levels of smoking behavior (*t* = −2.33, *p* < .05; Un *β* = −.28, SE = .19); at higher levels of future orientation, the negative link between relatedness satisfaction and smoking was stronger (Figures 1 and 2).

## 4. Discussion

We hypothesized that the FTP and basic psychological need satisfaction would be independently associated with healthy behaviors and that these variables would interact to predict greater levels of positive, and lower levels of negative, health behaviors. Our hypotheses were supported for positive health behaviors but only partially supported for negative health behaviors. Specifically, FTP but not basic psychological need satisfaction was negatively correlated with negative health behaviors, and we found no interaction between FTP and need satisfaction in predicting overall negative health behaviors. When we analyzed each negative health behavior individually, however, we found an interaction effect in predicting smoking, such that those with high basic psychological need satisfaction and high FTP were especially unlikely to smoke.

Our results support previous findings indicating a positive association between FTP and beneficial health outcomes (Henson et al., 2006). Moreover, our findings add to the growing evidence that FTP guards against negative health behaviors among college students (Adams, 2009). Similarly, greater fulfillment of BPNs, particularly relatedness, was associated with more positive health behaviors, which is consistent with past research (Vlachopolous & Michailidou, 2006).

Contrary to our hypothesis, BPN fulfillment was not associated with fewer negative health behaviors. Individuals who reported fulfillment of their psychological needs were more likely to pursue constructive behaviors, such as exercising, but they were not less likely to engage in negative health behaviors, such as eating sugary foods. Perhaps, this highlights important differences in the psychological processes at work during behavioral activation versus behavioral inhibition (Gray, 1990). The effectiveness of self-regulation depends in part on whether a person is pursuing goals and rewards (i.e. behavioral activation) or avoiding potential punishments (i.e. behavioral inhibition) (Carver & Scheier, 1999). Behavioral activation tends to be invigorating when BPNs are being fulfilled (Ryan & Frederick, 1997), whereas behavioral inhibition tends to involve controlling external factors that undermine psychological need fulfillment (Deci & Ryan, 2000).

Of particular interest, FTP and total BPN fulfillment interacted in predicting positive health behaviors. Individuals who reported a high FTP and high autonomy and relatedness, but not competence, were especially likely to report high positive health behaviors. The importance of autonomy in predicting adherence to positive health goals has been supported previously (Williams, Grow, Freedman, Ryan, & Deci, 1996) and our study indicates that this relationship is even stronger among individuals that are future-oriented versus those who are not. We also found that relatedness interacted with FTP, such that individuals high in both FTP and relatedness were especially likely to endorse positive health behaviors; this finding persisted across independent and combined analyses. In SDT research, relatedness is sometimes considered as a less central component to motivation, particularly in behaviors that occur in isolation (Deci & Ryan, 2000); however, relatedness may play a more influential role among college students than other age groups as friendship becomes increasingly important during individuals' transition into young adulthood (Larson, Richards, Moneta, Holmbeck, & Duckett, 1996). Participants in our sample who engaged in positive health behaviors may have done so with friends (e.g. exercising together), which can augment feelings of relatedness and improve commitment to positive health practices, particularly among individuals that are highly future-oriented. For instance, researchers have found that social support can improve the activation of and commitment to exercise routines (King & Frederiksen, 1984; Resnick, Orwig, Magaziner, & Wynne, 2002).

Inconsistent with our hypothesis, none of the BPNs interacted with FTP in predicting general negative health behaviors; however, when we examined smoking tobacco individually, autonomy, relatedness, and overall need fulfillment further strengthened the negative relationship between FTP and smoking. This is consistent with an intervention study that found autonomy supportive framing significantly improved smoking cessation rates (Williams et al., 2006). Motivational self-choice to avoid smoking may increase success, particularly for individuals who tend to consider and care about their future. Our data also suggest that satisfaction of one's need for relatedness predicts greater success in avoiding smoking among more future-oriented individuals, which is consistent with the longitudinal research demonstrating the importance of social support in smoking cessation and abstinence (Lawhon, Humfleet, Hall, Reus, & Munoz, 2009). As such, smoking cessation interventions should, whenever possible, integrate interpersonal elements as treatment strategies, such as establishment of rapport and involvement of family or peers. In health care settings, such efforts might be conducted effectively by nurses or behavioral health consultants (Blount, 2003).

Contrary to our expectations, there was no significant interaction between competence and FTP in the prediction of positive health behaviors. Potentially, for young adults or college students, the positive health behaviors we assessed, including eating well, exercising, and obtaining adequate sleep, are not associated with feelings of competence, as they may be considered somewhat normal or routine. As such, these behaviors may also be somewhat impervious to the effects of FTP. For college students, competence, as it relates to future perspective, may be tied to other aspects of life such as the pressure to perform academically (Macgeorge, Samter, & Gillihan, 2005).

### 4.1. Limitations

Our study is limited by the fact that it uses cross-sectional data. Although we found expected associations among our study variables, we do not know if FTP and BPN fulfillment lead to changes in health behaviors or if they are themselves contingent on health behaviors. Future research that is longitudinal in nature, or employs interval contingent sampling, is necessary and called for to better understand the causal nature of these interrelationships and to test and extend our hypotheses. Nonetheless, much empirical and theoretical work supports our presumed sequence of causation (Adams & Nettle, 2009; Deci & Ryan, 2002). Our study is also limited by the use of a predominantly White college student sample, which may not generalize to other age and ethnic groups; future research is necessary to extend our findings to more diverse samples. Furthermore, although we covaried age, it is important to recognize that young adults may have a particular lack of adeptness at envisioning the future and enacting consequent positive health behaviors based on a FTP (Wills, Sandy, & Yaeger, 2001). Therefore, replication is required to substantiate our findings and should be expanded to include other age groups. Despite using a psychometrically supported measure of FTP, recent research suggests that there may be additional aspects of future-oriented thinkers not captured by the ZTPI, including individual perceptions of how far into the future one should consider (Husman & Shell, 2008); therefore, a more comprehensive assessment of temporal perspectives is necessary in health research.

Our findings may have implications for designing interventions to improve health-related behaviors among college students. Given the importance of health behaviors to overall physical and psychological health (Mokdad et al., 2004), bolstering psychological dispositions that improve health behaviors may be a beneficial addition to treatments that students are already receiving, such as psychotherapy or primary care or even education. As an example, clinicians already employing Motivational Interviewing (MI), an evidence-based approach to augmenting motivation for engaging in healthy behaviors and avoiding risky health behaviors (Anstiss, 2009), may find additional benefit from promoting future-mindedness in their clients and by ensuring that treatment strategies are designed to support the patient's power to choose (autonomy), the patient's need for competence by encouraging challenging yet achievable goals, and the patient's need for relatedness by encouraging involvement of friends and family (Sirois & Hirsch, 2013). Indeed, some preliminary work has been conducted by linking the principles of change advocated in MI with the theoretical framework of SDT, which suggests that MI provides some of the social–environmental facilitating factors that promote adaptive growth (Markland, Ryan, Tobin, & Rollnick, 2005), and multiple intervention studies have demonstrated the outcome effectiveness of supporting BPNs in the context of occupation, education, sports, and health care (Deci & Ryan, 2008). In addition, preliminary trials suggest that time perspective can be modified through brief, targeted interventions and those subsequent changes in time perspective predict improved health behaviors (Hall & Fong, 2003). In the future, development and manualization of temporally based interventions that are focused on motivational enhancement are called for and should be tested in a randomized-controlled trial to determine their efficacy.

In conclusion, our study shows that future orientation and satisfaction of one's BPNs among college students are related to healthy behaviors and that an interaction exists such that being future-minded and satisfied in one's needs is especially predictive of engaging in positive health behaviors. Having a future-oriented perspective and feeling competent, supported in social relationships, and choiceful in one's actions is associated with behaviors that may help to prevent devastating health conditions such as obesity, diabetes, and cancer. Future research is needed to assess the additional and more narrowly defined negative health behaviors in order to clarify the role of future orientation and basic psychological need satisfaction in avoiding risky health behaviors.
